# Real-world deployment of a fine-tuned pathology foundation model for lung cancer biomarker detection

**DOI:** 10.1038/s41591-025-03780-x

**Published:** 2025-07-09

**Authors:** Gabriele Campanella, Neeraj Kumar, Swaraj Nanda, Siddharth Singi, Eugene Fluder, Ricky Kwan, Silke Muehlstedt, Nicole Pfarr, Peter J. Schüffler, Ida Häggström, Noora Neittaanmäki, Levent M. Akyürek, Alina Basnet, Tamara Jamaspishvili, Michel R. Nasr, Matthew M. Croken, Fred R. Hirsch, Arielle Elkrief, Helena Yu, Orly Ardon, Gregory M. Goldgof, Meera Hameed, Jane Houldsworth, Maria Arcila, Thomas J. Fuchs, Chad Vanderbilt

**Affiliations:** 1 Windreich Department of AI and Human Health, Icahn School of Medicine at Mount Sinai, New York, NY USA; 2 Hasso Platner Institute at Mount Sinai, Icahn School of Medicine at Mount Sinai, New York, NY USA; 3 Department of Pathology and Laboratory Medicine, Memorial Sloan Kettering Cancer Center, New York, NY USA; 4 Scientific Computing and Data, Icahn School of Medicine at Mount Sinai, New York, NY USA; 5 Department of Pathology, Molecular and Cell-Based Medicine, Icahn School of Medicine at Mount Sinai, New York, NY USA; 6 Institute of Pathology, Technical University of Munich, Munich, Germany; 7 Munich Data Science Institute, Technical University of Munich, Munich, Germany; 8 Department of Electrical Engineering, Chalmers University of Technology, Gothenburg, Sweden; 9 Institute of Clinical Sciences, University of Gothenburg, Gothenburg, Sweden; 10 Department of Clinical Pathology, Sahlgrenska University Hospital, Gothenburg, Sweden; 11 Department of Laboratory Medicine, University of Gothenburg, Gothenburg, Sweden; 12 Department of Hematology and Oncology, SUNY Upstate Medical University, Syracuse, NY USA; 13 Department of Pathology and Laboratory Medicine, SUNY Upstate Medical University, Syracuse, NY USA; 14 Tisch Cancer Institute, Icahn School of Medicine at Mount Sinai, New York, NY USA; 15 Department of Hematology–Oncology, University of Montreal Hospital Centre, Montreal, Quebec Canada; 16 Cancer Axis, University of Montreal Hospital Research Centre, Montreal, Quebec Canada; 17 Department of Medicine, Memorial Sloan Kettering Cancer Center, New York, NY USA; 18 Department of Medicine, Weill Cornell Medical College, New York, NY USA

**Keywords:** Predictive markers, Non-small-cell lung cancer, Machine learning

## Abstract

Artificial intelligence models using digital histopathology slides stained with hematoxylin and eosin offer promising, tissue-preserving diagnostic tools for patients with cancer. Despite their advantages, their clinical utility in real-world settings remains unproven. Assessing EGFR mutations in lung adenocarcinoma demands rapid, accurate and cost-effective tests that preserve tissue for genomic sequencing. PCR-based assays provide rapid results but with reduced accuracy compared with next-generation sequencing and require additional tissue. Computational biomarkers leveraging modern foundation models can address these limitations. Here we assembled a large international clinical dataset of digital lung adenocarcinoma slides (*N* = 8,461) to develop a computational EGFR biomarker. Our model fine-tunes an open-source foundation model, improving task-specific performance with out-of-center generalization and clinical-grade accuracy on primary and metastatic specimens (mean area under the curve: internal 0.847, external 0.870). To evaluate real-world clinical translation, we conducted a prospective silent trial of the biomarker on primary samples, achieving an area under the curve of 0.890. The artificial-intelligence-assisted workflow reduced the number of rapid molecular tests needed by up to 43% while maintaining the current clinical standard performance. Our retrospective and prospective analyses demonstrate the real-world clinical utility of a computational pathology biomarker.

## Abstract (web summary)

In a silent trial involving 197 patients with lung cancer over a span of 4 months, a fine-tuned pathology foundation model was able to identify the presence of *EGFR* mutations with high accuracy, potentially preventing further genetic testing in 43% of cases.

## Main

Lung adenocarcinoma (LUAD) is the most prevalent form of lung cancer and has been found to have multiple somatic mutations in kinase genes, among which *EGFR* is the most prevalent, that are treated with tyrosine kinase inhibitor (TKI) therapy^1^. Because tumors with *EGFR* mutations are treated with *EGFR*-specific TKIs, accurate *EGFR* testing is necessary for patients to receive the correct first-line therapy. Clinical testing guidelines provide guidance for the standard of care for molecular testing in lung cancer, and *EGFR* testing is a requirement for patients with advanced-stage LUAD, that is, stage IB or higher^2,3^. Despite these clear recommendations, *EGFR* testing is not performed on 24–28% (refs. ^4,5^) of lung cancer cases in the USA. The reason for the discrepancy between clearly published guidelines and actual clinical practice is not well understood but is probably related to technical hurdles in obtaining and processing samples for testing. Genomic sequencing, including targeted *EGFR* assays, is even less common in many regions of the world^6^. Given the high prevalence of *EGFR* mutation in LUAD, the lack of *EGFR* testing results in tens of thousands of patients worldwide receiving suboptimal therapy for *EGFR*-mutated LUAD every year.

Even in well-resourced centers that have adopted standards for universal *EGFR* testing for lung cancer, many samples fail to be assessed for *EGFR* status owing to insufficient material being available from diagnostic biopsies. Lung biopsies are inherently minute given the challenge of safely acquiring lung tissue. The number of tissue-based tests necessary for a proper diagnosis and to obtain comprehensive biomarker testing is large and ever-expanding. In this setting, a standard lung cancer diagnostic biopsy workup includes standard hematoxylin and eosin (H&E) sections, PDL1 immunohistochemistry (IHC), diagnostic IHC (for example, TTF-1, p40 and so on), *ALK* fusion IHC, rapid *EGFR* testing and comprehensive genomic sequencing. Turnaround times (TATs) are a notable challenge for the treatment of LUAD, especially for comprehensive sequencing with next-generation sequencing (NGS), which has a TAT of approximately 2–3 weeks from the date of the biopsy. Following guidelines, first-line therapy is typically not given until *EGFR* mutation status is known because (1) *EGFR* mutant tumors benefit from first-line TKI rather than chemotherapy/immunotherapy and (2) *EGFR* results further inform the likelihood of response to immune-based therapies. Rapid TAT testing has been developed and implemented to overcome this fundamental limitation of NGS. Owing to the targeted nature of rapid tests, they fail to detect less common *EGFR* variants, including many *EGFR* exon 20 insertions, uncommon exon 19 deletions and dinucleotide mutations resulting in common missense mutations (for example, p.L858R)^7,8^. This results in a technical sensitivity of 85–90% and a negative predictive value (NPV) of 90–95% in our experience, which means that 5–10% of samples that screen negative for *EGFR* mutations actually harbor a targetable mutation and would receive the incorrect first-line therapy^7,8^.

Computational methods to detect *EGFR* mutations that can be deployed with little cost, rapid TAT and automated implementation while preserving tissue for comprehensive genomic sequencing have the potential to greatly improve the clinical workflow for lung cancer diagnostic biopsies. Such a method has the potential to increase the detection of *EGFR* mutant lung cancer and reduce the number of suboptimal treatment regimens. Detecting mutations directly from H&E slides, if adequate performance characteristics are achieved, offers a unique opportunity to overcome many of the limitations of current clinical sequencing protocols for *EGFR*. A computational *EGFR* mutation assessment would use as substrate only the digitized pathology slides from the diagnostic H&E biopsy. A result could be reported with little cost and no physical processing. Such technology can also produce results immediately, which allows the results to inform all other downstream decisions.

Prior studies have shown that molecular biomarkers for somatic mutations can be predicted directly from routine H&E slides^9,10^. In the context of *EGFR* prediction in LUAD, the seminal work of Coudray et al.^11^ reported early results on the value of histology for this task. Despite the promising reported test area under the curve (AUC) of 0.826 (0.628–0.979), their result was based on a limited held-out test set that accounts for the wide 95% confidence interval. The model also required manual delineation of tumor boundaries before analysis, putting into question their clinical relevance. More recently, we and others have built highly effective *EGFR* detection models with modern weakly supervised techniques and convolutional neural network-based feature extraction encoders that can detect *EGFR* mutations with high accuracy on internal datasets^12,13^. Despite the evidence that *EGFR* mutational status can be predicted with reasonable accuracy from pathology slides, and despite the potential positive impact to patient care, no significant progress has been made toward a clinical implementation of these technologies.

**Fig. 1:**
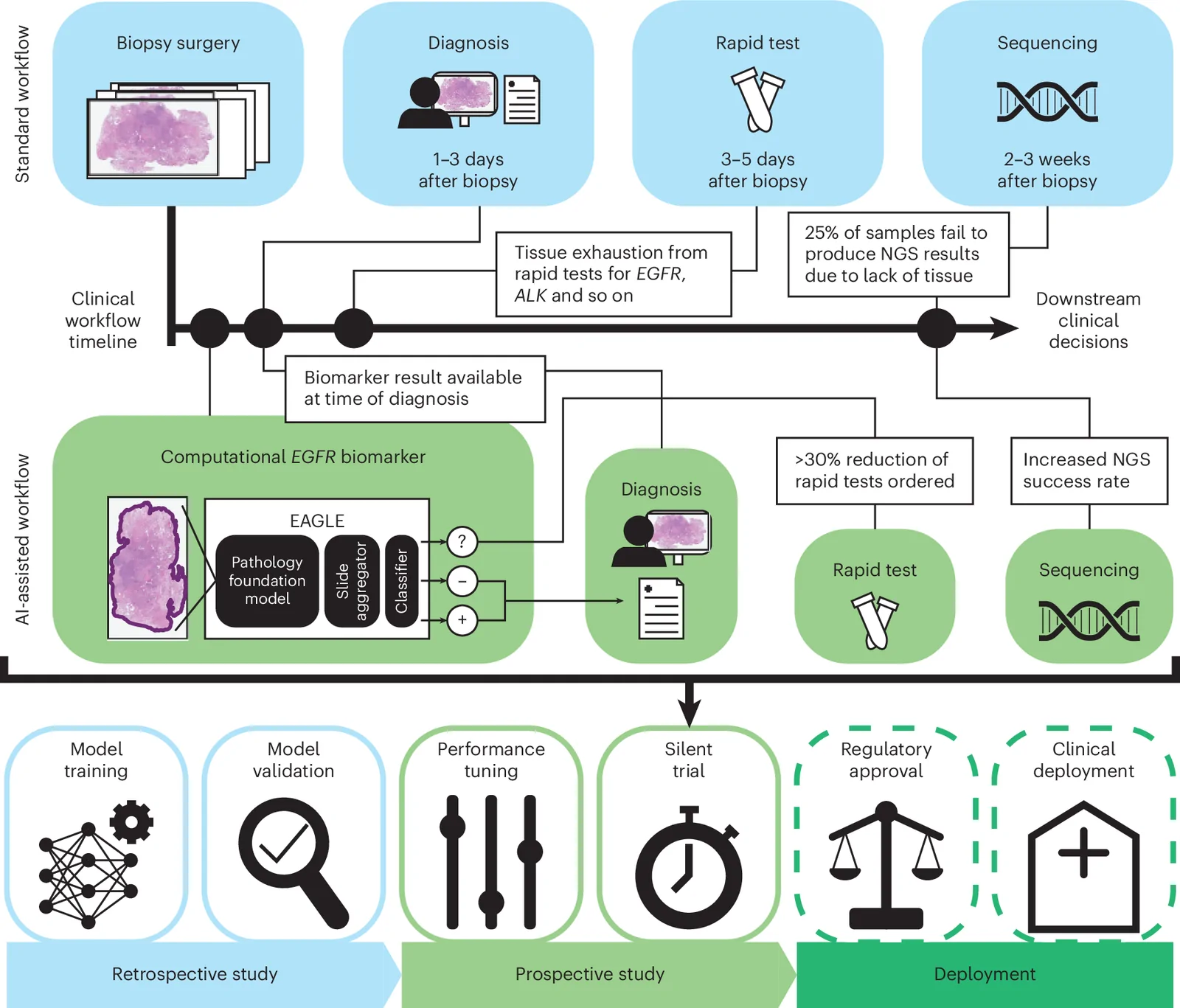
Clinical implementation of a computational *EGFR* biomarker. In the standard clinical workflow for patients with LUAD, rapid tests for *EGFR* and other biomarkers are performed, reducing the tissue available for NGS and leading to up to one-quarter of the cases being unsuitable for NGS. By contrast, the clinical application of the proposed *EGFR* biomarker will allow a drastic reduction in the number of cases unsuitable for NGS. As soon as slides are digitized, the computational biomarker can be calculated and may be available to the pathologist before they review the case and sign it out. Based on the model’s outputs, the rapid test may be avoided, increasing the tissue available for NGS.

In this work, we develop EAGLE (EGFR AI Genomic Lung Evaluation) and demonstrate its clinical utility as an H&E-based computational biomarker. EAGLE was designed to predict the *EGFR* mutational status from diagnostic biopsies of patients with LUAD, enhancing the standard molecular workflow (Fig. 1). Compared with the traditional workflow, the AI-assisted screening precludes rapid testing in a substantial amount of cases while maintaining overall high screening performance. It is important to note that samples that are screened positive still require NGS-based testing.

To achieve this milestone, we gathered a large clinical LUAD cohort, including samples from five national and international institutions, allowing us to train and evaluate our algorithm on digitized slides displaying the broad technical and biological variability expected from real clinical deployment. The system was trained by fine-tuning a state-of-the-art pathology foundation model on 5,174 slides from the Memorial Sloan Kettering Cancer Center (MSKCC). To assess the robustness of the model in detail and prove its generalization across institutions and scanners, we validated it on 1,742 internal slides from MSKCC and on external test cohorts comprising 294 slides from the Mount Sinai Health System (MSHS), 95 slides from Sahlgrenska University Hospital (SUH), 76 slides from the Technical University of Munich (TUM) and 519 slides from The Cancer Genome Atlas (TCGA).

To bridge the gap between proof of concept and clinical implementation, we performed a silent trial and deployed the model in real time to simulate its performance in a real-world setting. The prospective nature of this experiment demonstrates that EAGLE performs at the expected level on novel cases and is suitable for clinical implementation. We prove that the application of EAGLE can effectively reduce the need for rapid testing of *EGFR* without sacrificing the performance characteristics of the mutation screening process, with important ramifications for treatment decisions as we will discuss in detail. Finally, the data gathered from this experiment will be utilized to gain regulatory approval for use of EAGLE in clinical practice.

## Results

### Idylla performance and rapid test benchmarking

To assess the clinical performance of the *EGFR* rapid test assay, we collected Idylla test results for all patients with LUAD undergoing diagnostic workflow at MSKCC who also had a successful completion of MSK-IMPACT on the same tissue block from January 2022 to July 2024 (*N* = 1,685). We compared the rapid test results with the MSK-IMPACT NGS assay-derived ground truth and found that Idylla had a sensitivity of 0.918, a specificity of 0.993, a positive predictive value (PPV) of 0.988 and a NPV of 0.954 in the time period analyzed.

### Fine-tuning foundation model performance

**Fig. 2:**
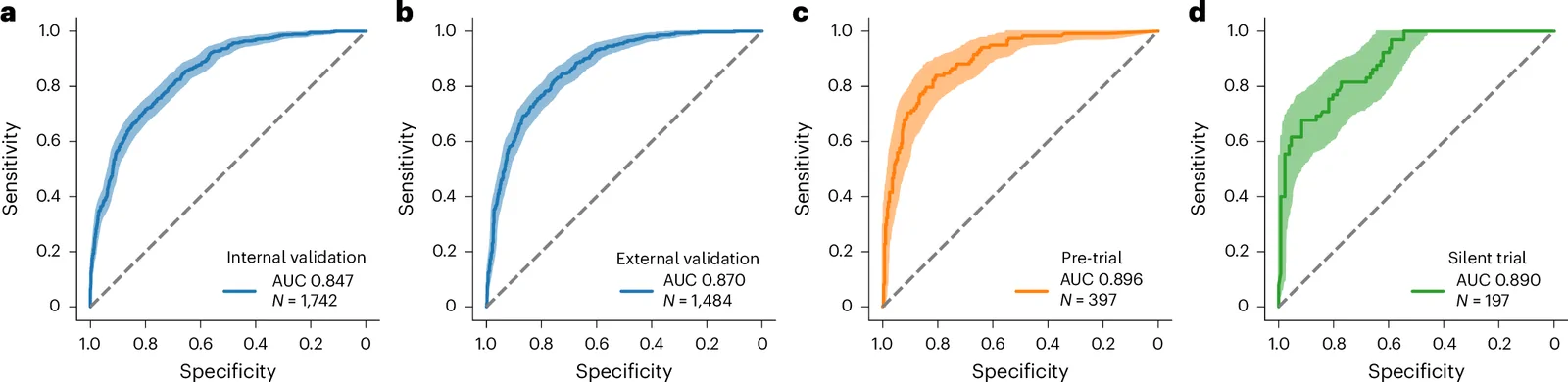
EAGLE performance on the internal and external cohorts. Receiver operating characteristic (ROC) curves and respective AUCs are presented. The ROC 95% confidence interval (shaded area) was calculated via bootstrapping with 1,000 iterations. **a**, Retrospective internal validation. **b**, Retrospective external validation. **c**, Retrospective pretrial cohort. **d**, Prospective silent trial cohort.

The performance of the trained model was assessed on the internal validation set that includes 1,742 slides. Overall, the model achieved an AUC of 0.847 (Fig. 2a and Supplementary Table 5). Model performance is more accurate in primary samples (AUC 0.90) than in metastatic specimens (AUC 0.75) as shown in Supplementary Fig. 2a. The analysis of metastasis location showed a pattern of differential performance (Supplementary Fig. 2b). These results are in support of the clinical application of EAGLE on primary samples.

We evaluated the model’s performance with respect to the amount of tissue present in the sample. We used tissue amount as a proxy for tumor amount. The tissue surface area was calculated on the basis of the tiles used for model inference. The distribution of tissue area over the internal validation set (Supplementary Fig. 3a) was divided into ten buckets by deciles. As metastatic samples contained on average less tissue, the analysis was done independently for primary and metastatic samples. The performance over tissue size is shown in Supplementary Fig. 3b and Supplementary Tables 6 and 7. We can observe a general trend of increasing performance as the area of the tissue being analyzed increases.

To ensure that the model does not systematically underperform for specific *EGFR* mutation variants, we compared the model’s probability scores across the mutation variants. We observed that the probability distributions across variants and compared with the overall distribution were nonsignificantly different (Supplementary Fig. 4 and Supplementary Table 8), highlighting how the model can detect all of the clinically relevant *EGFR* mutations. We also assessed the validation AUC restricted to each *EGFR* mutation variant (Supplementary Fig. 5) and found that the exon 19 T790M mutation had the highest AUC score, while the group of variants classified as ‘other’ had the lowest AUC score. All variants achieved AUC scores that were not significantly different from the overall AUC score, highlighting the robustness of EAGLE across variants.

### External validation is consistent with internal results

We assessed the performance of the proposed model on a variety of external cohorts from national and international institutions. The performance of the model on the external cohorts is consistent with the internal validation achieving an overall AUC of 0.870 on 1,484 slides (Fig. 2b), highlighting the generalization capacity of the proposed model. The MSHS cohort showed AUCs of 0.870 (*N* = 294), 0.877 (*N* = 241) and 0.884 (*N* = 259) for slides scanned with the Philips Ultrafast scanner, Aperio AT2 and Pramana, respectively. On the SUH cohort, the model achieved an AUC of 0.772 (*N* = 95), whereas on the TUM cohort the model achieved an AUC of 0.808 (*N* = 76). Finally, on the full TCGA cohort, the AUC was 0.860 (*N* = 519). Supplementary Fig. 1 and Supplementary Table 5 summarize these results.

Slides from the MSHS cohort were scanned using different scanner vendors. All the slides were scanned originally using the Philips Ultrafast scanner used for clinical operations. To assess the impact of digitization with different scanners, we rescanned the slides on the Aperio GT450 and Pramana scanners. A small number of slides could not be scanned on the Aperio and Pramana scanners owing to technical reasons. The number of slides scanned on three scanners and included in the scanner analysis is 224. We compared the pairwise linear correlations between the model scores for a slide scanned on two different scanners. We obtained Pearson coefficients of 0.828 (Philips versus Aperio), 0.832 (Philips versus Pramana) and 0.935 (Aperio versus Pramana), demonstrating the robustness of the proposed model across a variety of scanners (Supplementary Fig. 6 and Supplementary Table 9).

The TCGA cohort was extensively curated at the slide level with information about common artifacts present in that cohort. We analyzed whether these artifacts affected the performance of the model. For each type of artifact, we calculated the performance in terms of AUC when removing slides containing that artifact. We observed that the model was robust against all artifact types (Supplementary Fig. 7), highlighted by the stable AUC performance across the various stratifications. Further, by removing the slides that contained the most severe artifacts that obfuscated the tissue morphology, the AUC was increased from 0.860 on the overall cohort to 0.918.

### Silent trial supports the clinical use of EAGLE

**Fig. 3:**
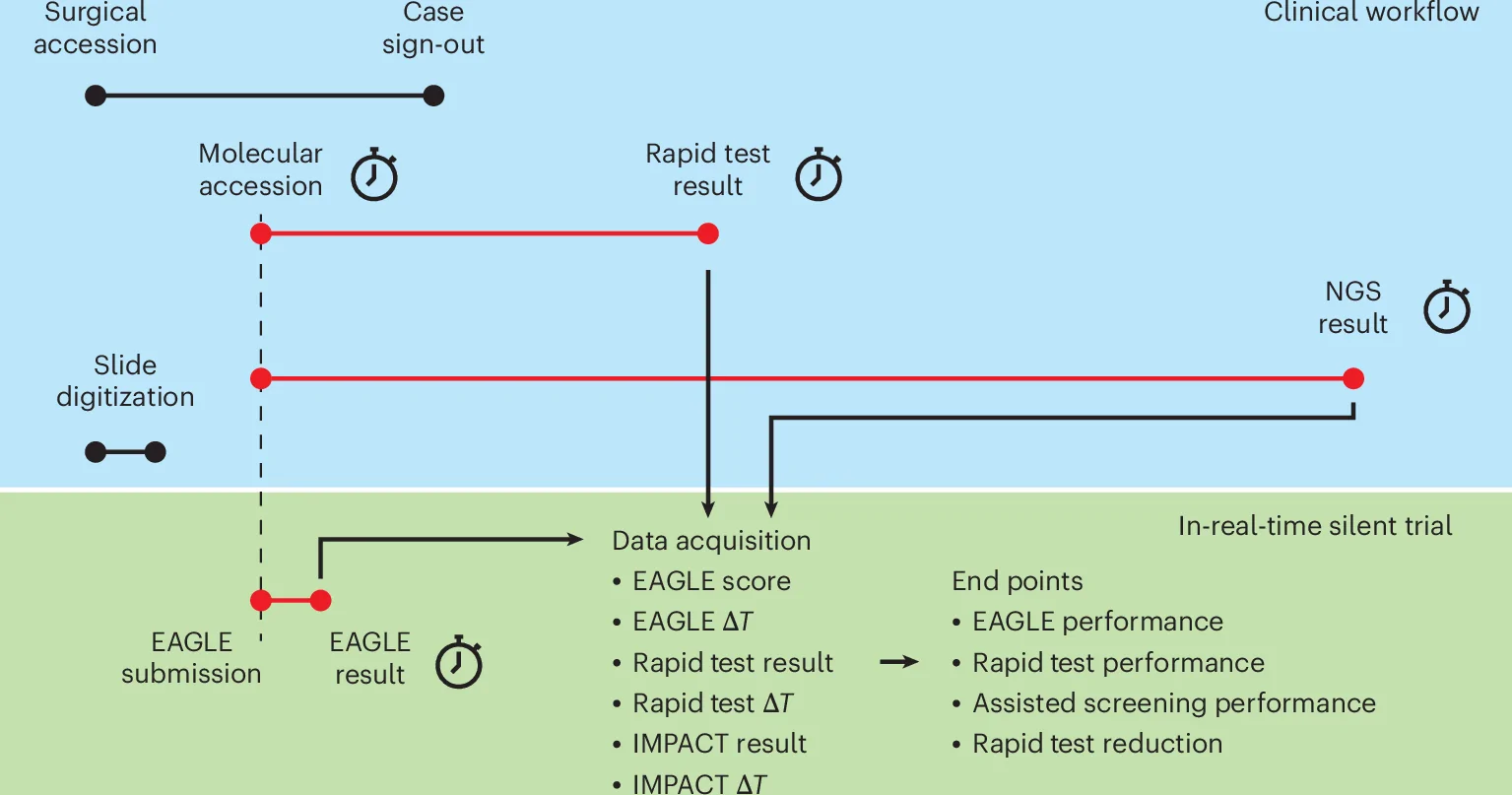
Silent trial workflow diagram. In blue, relevant components of the standard clinical workflow are shown along a timeline. Δ*T* indicates the time from molecular accession to the availability of a result. The silent trial components occurring in parallel with the clinical workflow are indicated in green.

Based on the internal and external validation results, we validated the clinical use of EAGLE for the prediction of *EGFR* mutations in LUAD samples extracted from the primary tumor site by running a silent trial at MSKCC. This experiment consisted of two stages. First, we leveraged the pretrial cohort to simulate the outcome of model deployment under various assistive strategies and chose appropriate model score thresholds. Second, using the threshold set, we run the in-real-time (IRT) silent trial capturing time and results from EAGLE, rapid test and MSK-IMPACT for analysis (Fig. 3).

We analyzed the pretrial cohort (*N* = 765) with EAGLE obtaining an overall AUC of 0.853, in line with the expected performance. In concordance with the internal validation, we observed that model performance is higher in primary samples (*N* = 374, AUC 0.896; Fig. 2c) than in metastatic specimens (AUC 0.760). Specific locations of metastases had particularly poor performances, lymph nodes (AUC 0.74) and bone (AUC 0.71). Other metastatic samples performed closer to the level seen on primary samples, liver (AUC 0.83) and brain (AUC 0.79). These results further support the deployment of EAGLE for primary samples.

**Fig. 4:**
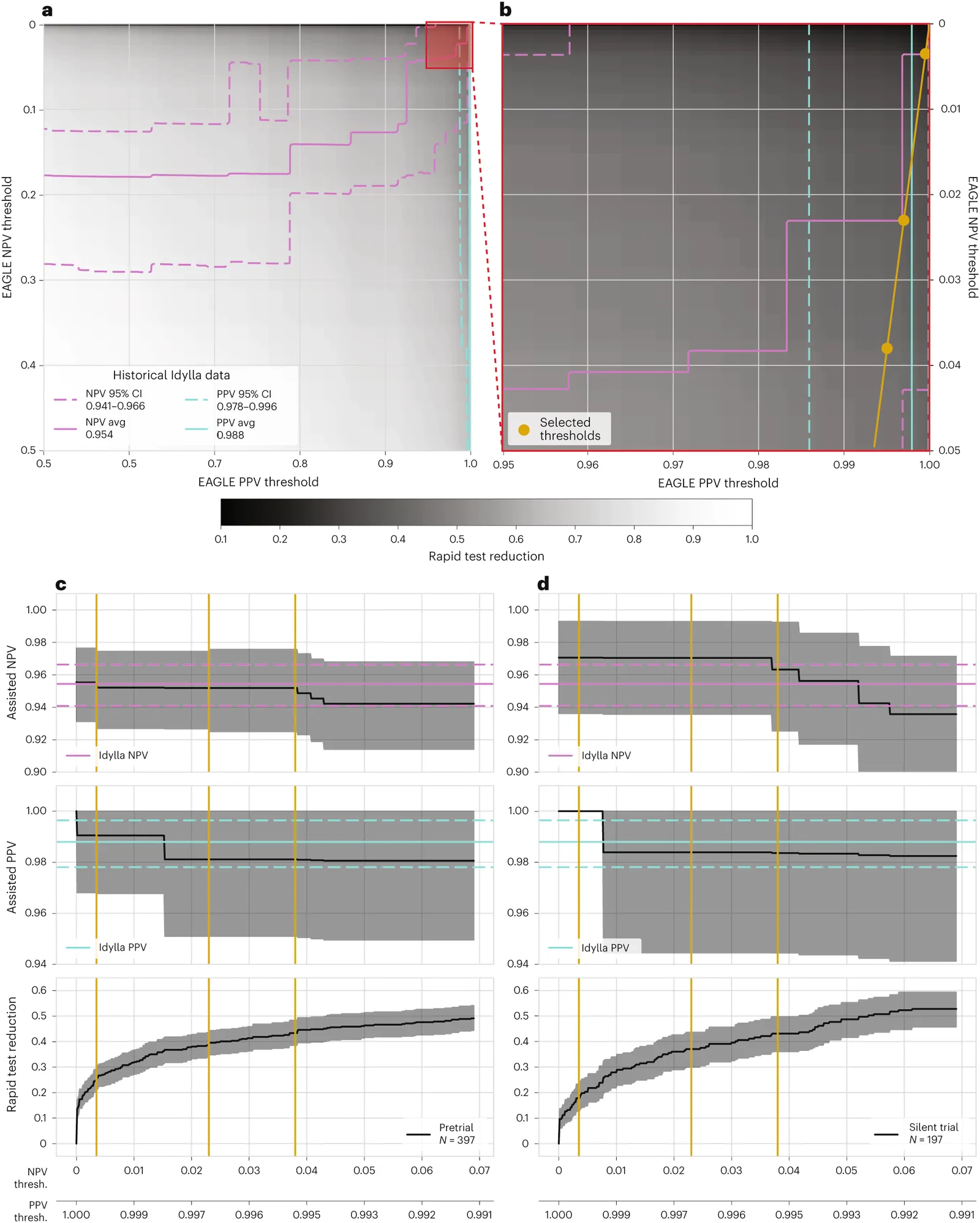
Pretrial tuning and silent trial results. In the AI-assisted *EGFR* screening, samples with EAGLE scores below the NPV threshold or above the PPV threshold can be spared from the rapid test. **a**, A heatmap of the reduction of rapid tests with isolines corresponding to the historical Idylla performance when modulating the NPV and PPV thresholds. Avg., average. The 95% confidence intervals (CI) were estimated via bootstrapping with 1,000 iterations. **b**, A zoomed-in area from **a** focusing on the top right corner where NPV and PPV are maximized. Threshold points are chosen within the Idylla noninferiority region with increasing levels of rapid test reduction. **c**, Pretrial deployment along the line established in **b** with the selected thresholds (thresh.) as vertical lines. The historical Idylla performance is shown with solid horizontal lines, and the dashed horizontal lines represent the 95% confidence intervals estimated via bootstrapping with 1,000 iterations. From top to bottom, NPV, PPV and rapid test reduction associated with AI-assisted workflow are presented. Shaded areas represent the 95% confidence interval estimated via bootstrapping with 1,000 iterations. **d**, A similar analysis as in **c** but for the silent trial cohort. The thresholds were chosen on the pretrial cohort and not on the silent trial cohort.

Based on the pretrial primary samples cohort, we investigated how EAGLE could be deployed as an aid to the current molecular workflow. In the current workflow, the polymerase chain reaction (PCR)-based rapid test is run on all LUAD samples. Under an artificial intelligence (AI)-assisted workflow, some of the samples may be spared from rapid testing based on the output of the EAGLE model. In the high-NPV domain (high-score regime), samples scored negative by EAGLE are probably true negatives and may not undergo rapid testing. On the other end of the spectrum, in the high-PPV domain (low-score regime), samples scored positive by EAGLE are probably true positives and may also be skipped. Given these two tunable parameters, we define the AI-assisted screening workflow as follows: (1) if the sample’s EAGLE score is below the NPV threshold, the sample is negative, no rapid test needed; (2) if the EAGLE score is above the PPV threshold, the sample is positive, no rapid test needed; (3) if the score is in between, the rapid test is performed for confirmation. To simulate deployment, we calculated various performance metrics associated with the AI-assisted screening when modulating the NPV and PPV thresholds. In Fig. 4a,b, we show as a heatmap the reduction of rapid tests and with isolines the historical performance of Idylla for NPV and PPV. The top right corner (thresholds 0, 1) constitutes the original rapid test performance without AI assistance (rapid test decrease equal to 0), whereas the bottom left corner (thresholds 0.5, 0.5) is equivalent to completely replacing the rapid test by EAGLE. Areas of high NPV and PPV intersect in the top right corner. We are interested in identifying points of the two-dimensional space where the assisted workflow is noninferior to the rapid test alone. We identified three points corresponding to three sets of thresholds with increasing levels of rapid test reduction, all within the noninferiority area. These points lie on a line that balances the joint optimization of NPV, PPV and rapid test reduction. Figure 4c shows the NPV, PPV and rapid test reduction associated with the AI-assisted workflow in the pretrial cohort along the path identified in Fig. 4b. The selected thresholds are also shown as vertical lines. With the most conservative threshold (0.004, 0.999), the assisted workflow is expected to yield 0.954 NPV, 0.991 PPV and 25% rapid test reduction. With the least conservative threshold (0.038, 0.995), the assisted workflow is expected to yield 0.952 NPV, 0.981 PPV and 43% rapid test reduction.

The assisted workflow was deployed IRT starting in May 2024 as part of a silent trial that lasted 4 months, from May to August 2024. On the samples analyzed (*N* = 197), EAGLE obtained an AUC of 0.890 (Fig. 2d) using the final MSK-IMPACT results as the ground truth, in line with the expected performance. Deployment of the AI-assisted workflow was evaluated at the three operating points defined for the pretrial cohort. In the most stringent setting, the assisted workflow achieved an NPV, PPV and rapid test reduction of 0.971, 1.0 and 18% respectively. In the least stringent setting, these metrics were equal to 0.963, 0.984 and 43%. The complete set of results are listed in Table 1. Figure 4d shows the continuous NPV, PPV and rapid test reduction of the AI-assisted screening in the silent trial. Overall, these results demonstrate the noninferiority of the AI-assisted workflow when deployed IRT in the clinical setting.

**Table 1:** Performance of the AI-assisted *EGFR* screening for the pretrial and silent trial cohorts

Cohort	Threshold		AI-assisted NPV		AI-assisted PPV		Test reduction	
	NPV	PPV	Average	95% CI	Average	95% CI	Average	95% CI
Pretrial	0.004	0.999	0.954	0.929–0.976	0.991	0.929–0.976	0.250	0.210–0.293
Pretrial	0.023	0.997	0.952	0.925–0.974	0.981	0.925–0.974	0.389	0.341–0.437
Pretrial	0.038	0.995	0.952	0.924–0.975	0.981	0.924–0.975	0.433	0.383–0.481
Silent trial	0.004	0.999	0.971	0.941–0.993	1.000	0.941–0.993	0.178	0.127–0.229
Silent trial	0.023	0.997	0.970	0.941–0.993	0.984	0.941–0.993	0.371	0.310–0.442
Silent trial	0.038	0.995	0.963	0.930–0.992	0.984	0.930–0.992	0.431	0.360–0.503

For each of the three deployment strategies identified in the pretrial cohort, the outcome of deployment is presented in term of NPV, PPV and reduction of rapid tests. Confidence intervals (CIs) were calculated using bootstrapping with 1,000 iterations.

We also analyzed the TATs of each test. We observed that EAGLE results are available with a median TAT from the time of molecular accession of 0.74 h (44 min), while Idylla has a median TAT of 48.78 h and MSK-IMPACT has a median TAT of 435.26 h (Supplementary Fig. 8a). These results are also documented in the context of the full case from surgical pathology accession to completion of MSK-IMPACT in Supplementary Fig. 8b. Supplementary Fig. 8b demonstrates that TAT relative to the surgical accession could be greatly improved if, for example, an order from the clinician for AI was provided.

### EAGLE model introspection

**Fig. 5:**
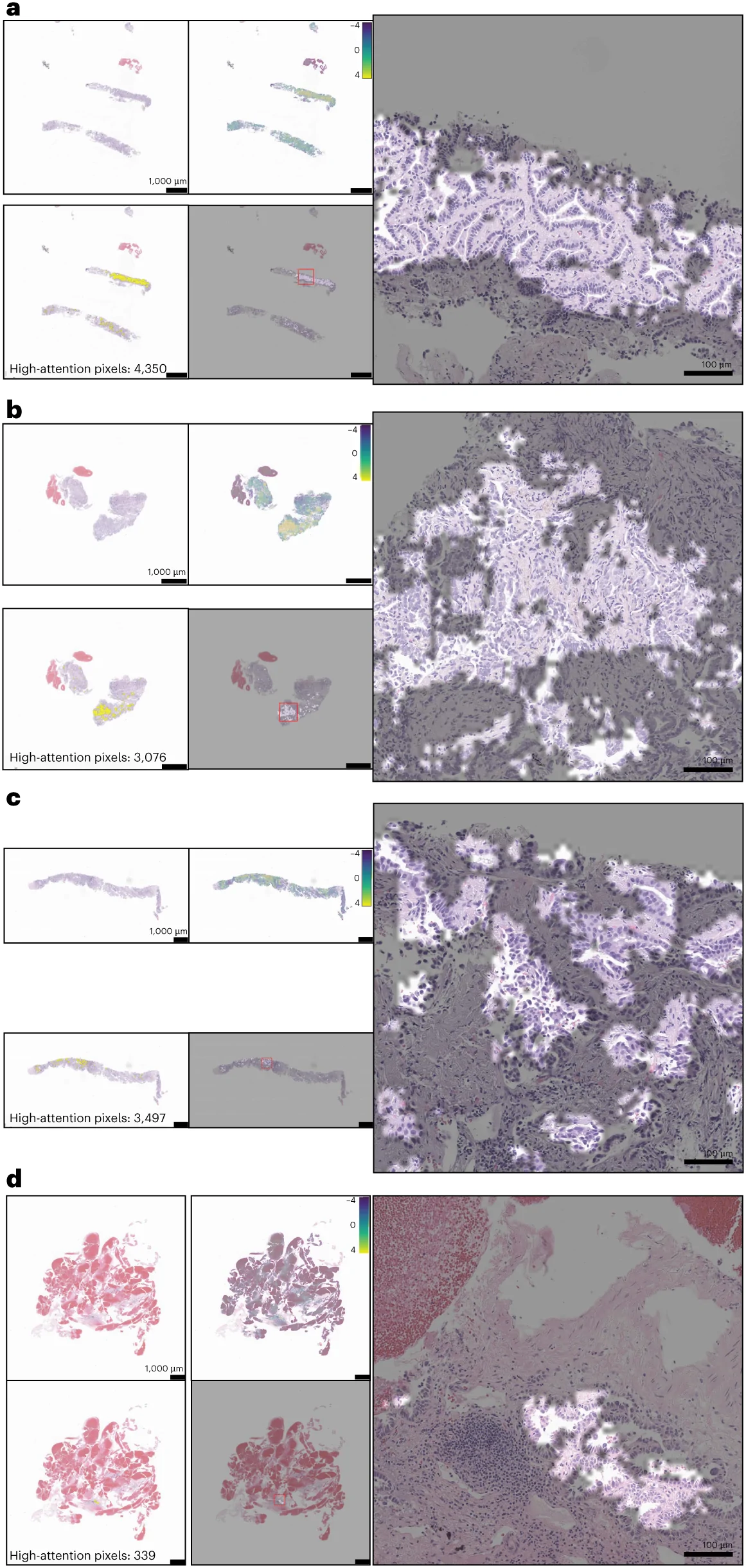
Model introspection using the attention scores from the aggregation function. As inference is deterministic, images are generated in one shot. Repeat inference generates identical image. **a**–**d**, Each figure is an example from the silent trial: true positive (**a**), true negative (**b**), false positive (**c**) and false negative (**d**). In each image, the top left is the thumbnail of the H&E WSI. The top middle contains an overlay of the thumbnail with the full spectrum of the attention mask from a score of −4 to 4 (see legend). The attention is the level to which the model is attending to the region on the image for making the decision of positive or negative (that is, does not indicate whether the model is interpreting the area as positive or negative, but only weighting). The bottom left has an overlay of the regions of the slide that have an attention score *>*3 (that is, high attention). The label at the bottom of panel is the quantity of pixels with high attention. The bottom middle has an inverted mask so that the non-high-attention regions are obscured. The red box in the panel indicates the region of the WSI that has the highest density of high-attention pixels. To the right is a high-resolution image of the portion of the slide highlighted by the red box in the prior panel.

The silent trial enables us to understand how our testing protocol performs in a real-world setting, including possible sources of false positive and false negative results. This ability for introspection is enhanced by generating image overlays to highlight the areas that are most attended to by the model (that is, ‘high attention’ areas). Figure 5 shows some examples of these attention maps. By analyzing the attention maps alongside the respective genomic profiles of each case, we came to several conclusions regarding false positives and false negatives. When evaluating cases that were predicted by EAGLE to have a high probability of being *EGFR* positive but that are negative by MSK-IMPACT, they had either (1) a biologically related mutation (for example, *ERBB2* exon 20 insertions) or (2) certain kinase activating events (for example, *MET* exon 14 skipping mutations and *ROS1* fusions). The case from Fig. 5c, for example, was predicted by the model to be positive for *EGFR* but has a *ERBB2* exon 20 insertion. False negative cases by the stated thresholds were too few to draw clear conclusions from the silent trial. To assess potential sources of false negatives, we analyzed *EGFR*-positive cases with EAGLE-predicted probability *<*0.5. These cases consisted of (1) cytology specimens lacking fragments with preserved tumor architecture, (2) biopsies with mostly blood and minimal fragments of tumor tissue, and (3) tumors with unusual morphologies for *EGFR* mutant LUAD (for example, tumors with high tumor-infiltrating lymphocytes and spindle-cell morphologies). The case from Fig. 5d has an EAGLE score of 0.43 but is positive for an *EGFR* mutation. The sample is largely blood and has very little tissue and almost no tumor, yet the attention mechanism correctly highlights the rare areas of tumor. Furthermore, the molecular results report the variant fraction as *<*5%. Thus, this sample is also borderline for molecular assessment owing to the relatively low tumor content. We hypothesize that manual interpretations by pathologists, with tools like the masks presented, could lower the error rate substantially.

## Discussion

The results presented in this study mark a significant milestone in the field of computational pathology, as this work demonstrates the real-world clinical-level performance of a computational pathology biomarker in a real-time setting. While many AI models have shown reasonable performance in cross-validation experiments on retrospective datasets, this study uniquely bridges the gap between model development and clinical deployment. The silent trial in which the model was applied prospectively to slides scanned in a live clinical setting represents a paradigm shift from controlled experimental conditions to true clinical utility. Importantly, in an IRT scenario, the model is applied to slides that did not exist when the model was finalized, ensuring a realistic test of the model’s ability to generalize to new, unseen data—a necessary step toward regulatory approval and use in clinical practice.

Most previous studies have concentrated on achieving high-performance metrics using retrospective, held-out test datasets^11–14^. While these results are promising, they have relied on carefully curated datasets with strict inclusion criteria and closely matched data distributions between training and test sets. Although retrospective studies facilitate performance optimization for a specific dataset, they do not adequately simulate how a model would perform in real-world clinical deployment. Furthermore, prior work has not demonstrated that these models can generalize to slides prepared in different laboratories, where variations in sample preparation, scanner hardware and staining protocols occur. In this study, we provide the first robust demonstration of cross-institutional and multiscanner performance for a challenging computational biomarker. In addition, the IRT study evaluates EAGLE under conditions of a real-world clinical settings, using routinely processed slides from a diverse patient population one would expect when utilized clinically. This silent trial establishes a critical benchmark for assessing the true clinical readiness of AI models in computational pathology.

The silent trial results support using EAGLE as a screening test complementary to existing rapid tissue-based tests, enhancing clinical genomics workflows. AI-assisted screening can reduce the number of costly, tissue-consuming rapid tests. Unlike rapid PCR-based assays, EAGLE detects all clinically relevant EGFR mutations. We propose EAGLE as a screening test, not a replacement for NGS sequencing; it efficiently rules out EGFR mutations and identifies likely positive cases. However, because it does not distinguish between mutations requiring different TKIs, sequencing confirmation is necessary before TKI therapy initiation.

The prospective nature of the silent trial distinguishes our study from prior work. EAGLE maintained high accuracy on real-time clinical samples, achieving an AUC of 0.890, consistent with internal and external validation, highlighting the model’s robustness and readiness for clinical implementation. In simulating clinical use, EAGLE predictions were available within a median of 44 min, substantially faster than rapid tissue-based tests (48 h) and comprehensive genomic sequencing (2–3 weeks). This rapid turnaround allows clinicians to make informed decisions sooner, potentially initiating therapies earlier and improving patient outcomes. The use of EAGLE would conserve biopsy material for comprehensive genomic testing, resulting in fewer test failures owing to insufficient material and the need for repeat biopsies.

Automated deployment delivers results directly to pathologists, enhancing interpretation within clinical contexts. Incorporating pathologists into the workflow could further improve model performance. The performance of the rapid molecular test, which requires a molecular pathologist’s interpretation, is enhanced as equivocal results are deferred until confirmation or refutation of the mutation is provided by NGS. A pathologist-in-the-loop workflow would similarly benefit clinical AI models.

In this regard, we have shown that analysis of model attention maps alongside histology and mutation profiles identified trends associated with false positives and negatives. Samples with minimal tumor architecture, specifically cytology samples, generally exhibit lower scores and attention. For these samples, different triage strategies (for example, additional quality check and separate models) may be necessary to deliver similar performance as larger biopsies. The false positives point to overlapping tumor morphologies in certain biologically similar mutations that activate kinase domains of protooncogenes.

A major advantage of computational methods is rapid inference, with median inference time of 68 s on a consumer-grade graphics processing unit (GPU). Currently, model inference is triggered by molecular accession, although slides are typically scanned several days earlier. Thus, if the clinical workflow involved an ‘AI service’ where the clinician could order the AI result along with the biopsy, the EAGLE result could be produced and reported even sooner.

This trial focused on deploying EAGLE in an institution routinely performing rapid EGFR testing. However, EAGLE offers greater efficiency gains in hospitals relying solely on NGS. In this setting, EAGLE would allow much earlier initiation of chemotherapy or immunotherapy for tumors screened negative. Conducting a separate silent trial in such settings would be valuable to understand how EAGLE could enhance such a workflow. We have shared code and model weights, enabling external laboratories to perform calibration studies and validate EAGLE for their specific clinical workflows.

Another key aspect of this work is the use of a foundation model for feature extraction, which enabled the development of a highly generalizable and robust *EGFR* prediction model. Foundation models, trained on large, diverse datasets, offer several advantages over traditional deep learning models, including improved transfer learning capabilities and feature representations that are adaptable to a wide range of tasks^14–16^. In this study, we fine-tuned a state-of-the-art pathology foundation model (Prov-GigaPath ViT-g) to achieve clinical-level performance for the specific task of *EGFR* mutation detection in LUAD.

The success of the foundation model approach is evident in the model’s ability to generalize across institutions, patient populations and scanning equipment, a challenge that has hindered the clinical translation of computational pathology models. This flexibility is crucial for real-world deployment, where variability in slide preparation and digitization is inevitable. The fine-tuning strategy used here further enhances the foundation model’s performance, yielding a significant improvement in AUC compared with previous studies using traditional convolutional neural networks. For instance, our model achieves a mean AUC of 0.861 across multiple external cohorts, surpassing the AUC of 0.826 reported by Coudray et al.^11^ on a smaller, retrospective dataset.

Although the results of this study are promising, there are several limitations that will be addressed in future work. First, the model’s performance was lower in metastatic samples compared with primary tumor samples, suggesting that additional data and refinement may be needed to optimize predictions in metastatic settings. Metastatic samples are inherently more heterogeneous and often display varied morphologies or morphologies distorted by the substrate of the organ metastasized to, which could affect the model’s ability to capture relevant features. Metastatic samples also are more commonly obtained after initial treatment, and there may be morphological divergence associated with prior therapy and molecular evolution of the tumor. Expanding the training set to include a greater diversity of metastatic samples and/or restricting deployment to metastatic sites where performance is proven will be needed for clinical deployment for metastatic samples.

Second, although the model was validated on a large external dataset and evaluated prospectively in a silent trial, further studies are needed to assess its impact on clinical decision-making and patient outcomes. A prospective interventional trial in which EAGLE’s predictions are used to guide clinical management would provide definitive evidence of its utility and may pave the way for updating guidelines to include EAGLE use in clinical settings.

Third, although this study focused exclusively on *EGFR* mutation prediction, the same approach could be extended to other clinically relevant biomarkers, such as *ALK*, *ROS1* and *RET* fusions. These molecular events are much less common, and accumulating the cases to properly train and validate a model, as we have with EAGLE, is more challenging and would require longer IRT trials. We are in the early stages of developing a similar model for *ALK* fusions and *MET* exon 14 skipping mutations. We also anticipate the development of models trained to evaluate more complex predictive tasks such as patient prognosis and treatment response prediction. As we and other researchers develop such models, the use of AI-based models for biomarker prediction may become a routine aspect of LUAD pathological evaluation.

The successful application of EAGLE in a real-time clinical setting demonstrates the potential of computational pathology models to transform traditional diagnostic workflows. With the advent of foundation models, computational pathology is poised to become a critical component of precision oncology, offering scalable, low-cost, automated and highly accurate solutions for a wide range of clinical tasks. Unlike traditional diagnostic tools, AI-based computational biomarkers are inherently digital, enabling remote deployment and democratization of advanced diagnostic capabilities to underserved regions worldwide. By reducing or eliminating the need for tissue-based testing and offering rapid, reproducible results, AI models like EAGLE can help to bridge gaps in care and ensure that all patients, regardless of geographic or economic barriers, have access to state-of-the-art diagnostic tools.

In conclusion, this study represents a successful demonstration of a real-time, clinically validated computational pathology model for *EGFR* mutation detection in LUAD. By leveraging the strengths of foundation models and validating the model in a prospective IRT setting, we have established a benchmark for clinical-level performance in computational pathology. The deployment of EAGLE has the potential to improve diagnostic efficiency, reduce tissue consumption and accelerate the adoption of AI in routine clinical practice. Future work should focus on expanding the model to include additional biomarkers and evaluating its impact on therapeutic outcomes in a prospective clinical trial. The insights gained from this study provide a roadmap for the integration of AI into clinical pathology and highlight the transformative potential of computational biomarkers for precision oncology.

## Methods

### Whole-slide images and sequencing datasets

This research study was approved by the respective institutional review boards at the Icahn School of Medicine at Mount Sinai (protocol 19-00951) and MSKCC (protocol 18-013). Informed consent was waived as per the institutional review board protocols. Participants were not compensated. Sex and/or gender was not considered in the study design as cohorts were generated as random samples of the patient population.

### MSKCC

The MSKCC cohort consists of 7,586 patients (7,996 slides or cases) diagnosed as LUAD from 2014 through 2024, divided into the training dataset (4,867 patients, 5,174 slides), validation dataset (1,640 patients, 1,742 slides), dataset for calibrating clinical threshold (764 patients, 765 slides, 397 primary) and the slides processed in real time for the silent trial (315 patients or slides, 197 primary). For the retrospective cohorts (training and validation), the last section taken from each formalin-fixed paraffin-embedded (FFPE) tissue block (after all unstained slides used for molecular sequencing have been cut) is stained with H&E. All samples, including cytology cell block samples, are part of the retrospective cohort. The calibrating and IRT datasets are evaluated using the diagnostic slides, including from cytology cell blocks, the first section from the block, before either the rapid test or genomic sequencing. All slides are digitized with a mix of Aperio AT2 (at 20× magnification) and GT450 (at 40× magnification) digital slide scanners from Leica Biosystems. All slides that were part of the standard clinical workflow were utilized. Thus, this study represents the full extent of biological and technical variability of the clinical setting. The cases for prospective sequencing are selected in real time by identifying LUAD samples for which both rapid EGFR genomic sequencing is ordered prospectively. Demographic and clinical information for this cohort is provided in Supplementary Table 1.

The ground truth for EGFR mutations is established using the MSK-IMPACT targeted genomic sequencing assay^17,18^, performed on the same tissue block from which the digital slide is created. MSK-IMPACT is a hybridization capture-based NGS assay routinely used to detect clinically relevant somatic mutations, copy number alterations and gene fusions across cancers. This assay screens for variants in up to 505 unique cancer-related genes, including EGFR, in all tumor types. All sequencing occurs in a Clinical Laboratory Improvement Amendments (CLIA)-certified laboratory, and each variant is reviewed by a board-certified molecular pathologist.

For LUAD samples, the standard clinical workflow includes rapid EGFR mutation testing via the Idylla platform. In this process, tissue is scraped directly from unstained slides and placed into a cartridge. The cartridge, once mounted onto the analyzer, facilitates both DNA extraction and all PCR reactions in a fully automated, self-contained manner. Output data are then uploaded to the vendor’s website, where the platform provides analysis and interpretation of common EGFR mutations. A board-certified pathologist interprets the results, which are subsequently reported in the clinical setting. For this study, we utilized the results as documented in the laboratory information system following pathologist review and interpretation.

### MSHS

For the MSHS samples, an H&E-stained section adjacent to the ones used for sequencing was reviewed for tumor cellularity and served as the source of imaging for the current study. In total, 294 slides from 287 patients were obtained and scanned using three scanner models: Philips Ultrafast (*N* = 294), Aperio GT450 (*N* = 241) and Pramana (*N* = 259). Some slides failed to be scanned on the Aperio and Pramana scanners. This cohort was entirely dedicated to testing the proposed system. Demographic and clinical information for this cohort is provided in Supplementary Table 2.

The MSHS cohort comprised LUAD specimens consecutively ascertained and subjected to molecular profiling during August 2018 to April 2021 as part of routine patient clinical care. Genomic DNA and RNA were extracted from eight to ten unstained FFPE tissue sections and then sent out for profiling using the Oncomine Comprehensive Assay v3 (ThermoFisher Scientific). Clinically relevant variants (single-nucleotide variants, indels, gain and fusions) were extracted from reports.

### SUH

Consecutive FFPE material of LUAD tumors (*N* = 95) was obtained through surgical resections (sublobar wedge resections or lobectomy), collected at the Department of Clinical Pathology, SUH, between 2017 and 2022. The glass slides were scanned with a 40× mode Nanozoomer S210 Digital Slide Scanner (Hamamatsu Photonics K.K.) with a resolution of 0.23 µm per pixel. Demographic and clinical information for this cohort is provided in Supplementary Table 3. For the SUH cohort, the Oncomine Focus Assay (Thermo Fisher Scientific), a targeted, multibiomarker assay that enables detection of hotspots, single-nucleotide polymorphisms, indels, copy number variations and gene fusions from DNA and RNA in a single workflow, was used and analyzed by Ion Torrent NGS systems (Thermo Fisher Scientific). This analysis covers variants across 52 major genes with frequent alterations in non-small-cell lung cancer (NSCLC).

### TUM

FFPE tissue sections of patients with LUAD (*N* = 41) were obtained at the Institute of Pathology of the TUM between 2019 and 2021. The patients’ tissue slides have been digitized using two slide scanners, Aperio AT2 at 0.5 µm per pixel (*N* = 38) and Aperio GT450Dx at 0.25 µm per pixel (*N* = 38), totaling 76 whole-slide images (WSIs). Demographic and clinical information for this cohort is provided in Supplementary Table 4.

For the molecular analysis, the TruSight Oncology 500 assay (Illumina) was used. This pan-cancer assay allows targeted-capture sequencing of 523 cancer-related genes at the DNA level and translocation detection of 50 driver fusion genes at the RNA level. Sequencing was performed on a NextSeq 550DX (Illumina) system using a NextSeq 500/550 High Output Kit v2.5 (300 Cycles). Data were processed and analyzed by the TruSight Oncology 500 Local App version 2.11.3, followed by an in-house pipeline using a second variant caller (Mutect2^19^) and ANNOVAR^20^ for annotation of the alterations. For DNA analysis, single-nucleotide variants, insertions and deletions, copy number variations, total mutation burden and microsatellite instability were calculated. For RNA analysis, putative gene fusion of around 50 fusion driver genes and RNA splice variants from *EGFR*, *AR* or *MET* (for example, *MET* exon 14 skipping) were explored.

### TCGA

The TCGA-LUAD is a well-characterized cohort of primary resection specimens that is part of the broader TCGA project^21^. TCGA-LUAD originally consisted of comprehensive genomic profiling of 230 resected LUADs by whole-exome sequencing (WES) and RNA sequencing. The cohort has subsequently been expanded to 585 unique cases with 582 undergoing WES. The corresponding 519 diagnostic digital slides were downloaded using the GDC Data Transfer Tool. An expert pathologist reviewed all samples, annotating the presence of different types of artifact on the slides: low-quality stain, blue and red saturation, blur, widespread tissue necrosis, freeze artifacts and severe artifacts that completely obscured the morphology of the sample (Supplementary Table 10). EGFR mutations from WES were clinically characterized using the OncoKB database^22^. Importantly, mutations outside of the EGFR kinase domain (exons 18–24) are not oncogenic and are excluded from the analysis. Oncogenic EGFR mutations are grouped into the common subtypes: (1) exon 19 deletions, (2) L858R, (3) exon 20 insertions, (4) T790M, and (5) other kinase domain mutations.

### EAGLE—EGFR prediction model

The proposed model consists of (1) a 1.1-billion-parameter vision transformer^23^ (ViT-g) that encodes high-resolution (20× magnification, 0.5 μm per pixel) 224-pixel patches into a 1,536-feature vector; (2) a gated multiple instance learning (MIL) attention (GMA) aggregator that integrates all encoded patches from a slide into a global slide-level feature representation; and (3) a linear classifier that outputs the probability of an EGFR mutation based on the input slide data.

During training, the encoder was initialized with Prov-GigaPath^16^, a public state-of-the-art pathology foundation model. The full model was then trained end to end using the parallelization strategy described by Campanella et al.^24^. In brief, to allow joint optimization of the encoder, aggregator and classifier, the encoding is parallelized across numerous processes to divide the GPU memory burden across several GPUs. A separate GPU receives the encoded images, aggregates them with GMA and produces the classification loss. During backpropagation, the gradients are directed to each process and synchronized. For each slide, 6,624 tissue patches were sampled at each training step and divided across 23 GPUs for encoding (96 patches per GPU). The patch encoding was done in 16-bit float precision to enable the use of larger image batches. Overall, the model was trained on 24 NVIDIA H100-80GB GPUs for 20 epochs in around 9.28 h. At inference time, the trained model can be run on a single GPU. For the silent trial, we deployed EAGLE with full floating-point precision using one NVIDIA RTX 3090 GPU with 26 GB. The time required to process a slide had a median of 68 s, making it suitable for real-time application in the clinical workflow. On lower-capacity hardware, the deployment of EAGLE is still possible by trading off memory consumption with inference speed.

### Real-time EGFR prediction clinical pipeline

Figure 3 illustrates the proposed IRT pipeline to identify and process WSIs of primary samples of LUAD specimens for EGFR prediction in a live, real-world, clinical setting in the context of a silent trial. MSKCC processes 90–110 NSCLC cases per month for which EGFR testing is clinically indicated. The proposed IRT pipeline automatically identifies slides that are scanned for molecular testing as well as the slides scanned from the same surgical pathology block for which molecular testing was ordered. Two watcher applications are run automatically on an hourly cadence to identify (1) which slides have been scanned and (2) which lung cancer cases are sent for molecular analysis. When a slide matches a molecular case of interest, the slide is transferred from the digital pathology system to the GPU compute infrastructure, and inference on the AI model is run immediately. This setup allows automated, real-time EGFR prediction. If two or more WSIs are scanned, the first scanned slide is used. During the course of the silent trial, several important data points were collected, in particular, the results from EAGLE, rapid testing and MSK-IMPACT. In addition, timestamps from key events were recorded: when the rapid test is accessioned (which triggers the execution of EAGLE), when the result from EAGLE is produced, when the rapid test result is generated and when the MSK-IMPACT test result is ready. Based on this information, we can assess the performance of the assisted screening pipeline consisting of EAGLE and the rapid test against the current workflow consisting of the rapid test alone.

### Software

The model was developed using pytorch (v.2.1.1+cu121). Software pipelines were built with Python (v.3.8.18).

### Reporting summary

Further information on research design is available in the Nature Portfolio Reporting Summary linked to this article.

## Online content

Any methods, additional references, Nature Portfolio reporting summaries, source data, extended data, supplementary information, acknowledgements, peer review information; details of author contributions and competing interests; and statements of data and code availability are available at https://doi.org/10.1038/s41591-025-03780-x.

## Supplementary information

Supplementary Information Supplementary Figs. 1–8 and Tables 1–9.

Reporting Summary

## Data Availability

Clinical datasets, digital slides and genomics (Memorial Sloan Kettering Cancer Center, Mount Sinai Health System, Technical University Munich and Sahlgrenska University Hospital) used for this study are not made available in accordance with institutional policies. The TCGA dataset, images and genomics are available via GDC Portal at https://portal.gdc.cancer.gov/projects/TCGA-LUAD. Clinical annotation of variants was performed using OncoKB API (https://www.oncokb.org/api-access). Model checkpoints are publicly available via HuggingFace at https://huggingface.co/MCCPBR/EAGLE.

## Code availability

Code used in this work is available via GitHub at https://github.com/chadvanderbilt/EAGLE.
