# Supplementary material for: Real-world deployment of a fine-tuned pathology foundation model for lung cancer biomarker detection
Source: Nat Med. 2025 Jul 9;31(9):3002–10. doi: 10.1038/s41591-025-03780-x (PMC12443599; doi:10.1038/s41591-025-03780-x)
Supplement: Supplementary file 1 — Supplementary Figs. 1–8 and Tables 1–9. [file 41591_2025_3780_MOESM1_ESM.pdf]

nature medicine

Supplementary information https://doi.org/10.1038/s41591-025-03780-x

Real-world deployment of a fine-tuned pathology foundation model for lung cancer biomarker detection

In the format provided by the authors and unedited

Supplementary Material

Cohort Descriptions

	Category	Training Dataset	Validation Dataset	Calibrating Threshold Dataset	In Real Time Dataset
Sex	Female	3129 (64.29%)	1032 (62.93%)	471 (61.65%)	210 (66.67%)
	Male	1738 (35.71%)	608 (37.07%)	293 (38.35%)	105 (33.33%)
Smoking History (NLP)	Former/Current Smoker	3291 (67.62%)	1093 (66.65%)	487 (63.74%)	215 (68.25%)
	Never	1466 (30.12%)	501 (30.55%)	259 (33.9%)	93 (29.52%)
	Unknown	110 (2.26%)	46 (2.8%)	18 (2.36%)	7 (2.22%)
Race	Asian-Far East/Indian Subcontinent	556 (11.42%)	164 (10.0%)	89 (11.65%)	41 (13.02%)
	Black or African American	259 (5.32%)	88 (5.37%)	51 (6.68%)	16 (5.08%)
	Native American-Am Ind/Alaska	6 (0.12%)	1 (0.06%)	0 (0.0%)	0 (0.0%)
	Native Hawaiian or Pacific Islander	3 (0.06%)	1 (0.06%)	1 (0.13%)	0 (0.0%)
	White	3772 (77.5%)	1294 (78.9%)	553 (72.38%)	248 (78.73%)
	Unknown or Other	250 (5.14%)	84 (5.12%)	68 (8.9%)	10 (3.17%)
Stage (Highest Recorded)	Stage 1-3	2621 (53.85%)	892 (54.39%)	485 (63.48%)	132 (41.9%)
	Stage 4	2002 (41.13%)	661 (40.3%)	243 (31.81%)	43 (13.65%)
	Unknown	244 (5.01%)	87 (5.3%)	36 (4.71%)	140 (44.44%)
Age at Diagnosis	years, mean [95% Confidence Interval]	65.67 [65.37, 65.97]	65.22 [64.70, 65.74]	68.57 [67.81, 69.33]	68.63 [67.53, 69.73]
Source Material	Surgical	4070 (83.62%)	1404 (85.61%)	620 (81.15%)	258 (81.90%)
	Cytology	797 (16.38%)	236 (14.39%)	144 (18.85%)	57 (18.09%)
Sample Type	Primary	3104 (63.79%)	1038 (63.29%)	480 (62.83%)	225 (71.43%)
	Metastatic	1762 (36.21%)	602 (36.71%)	284 (37.17%)	90 (28.57%)

Supp. Table 1 Clinical Characteristics of MSKCC Cohorts Used for Training, Validating, Calibrating, and Real-Time Analysis. Values for Sex, Smoking History, Race, and Stage are obtained from internal CbioPortal instance (obtained October 10, 2024). Smoking status is obtained by natural language processing (NLP) of clinical notes from the patients charts. Stage is highest recorded stage in the patient's clinical history at the time data is obtained, not the stage at the time of diagnosis.

Supp. Table 2 MSHS Cohort Description.

	Slides	*EGFR*+ %	Patients
Overall	294	35.0	287
Sex			
NA	56	16.1	55
Female	133	44.4	130
Male	105	33.3	102
Race			
NA	66	16.7	65
White	106	35.8	105
Asian	33	54.5	32
Black	40	35.0	39
Other	49	44.9	46
Smoking			
NA	43	30.2	
Never	80	65.0	
Past	136	25.5	
Current	35	5.7	
Age at Diagnosis			
NA	56	16.1	
30-40	4	75.0	
40-50	8	37.5	
50-60	26	19.2	
60-70	59	44.1	
70-80	83	42.2	
80-90	54	37.0	
90-100	4	50.0	
Stage			
NA	173	36.4	
1	61	41.0	
2	8	50.0	
3	16	0.0	
4	36	30.6	

Supp. Table 3 SUH Cohort Description.

	Slides/Patients
Overall	95
EGFR Status	
EGFR mut	58
EGFR wt	37
Sample Type	
Primary	51
Metastatic	44
Sex	
Female	53
Male	42
Age at Diagnosis	
20-30	0
30-40	3
40-50	4
50-60	4
60-70	20
70-80	48
80-90	8
90-100	0
NA	8

Supp. Table 4 TUM Cohort Description.

	Slides/Patients
Overall	76/41
EGFR Status	
EGFR mut	23
EGFR wt	18
Sample Type	
Primary	27
Metastatic	14
Sex	
Female	17
Male	24
Scanner	
AT2	38
GT450Dx	38

Validation and Test Results

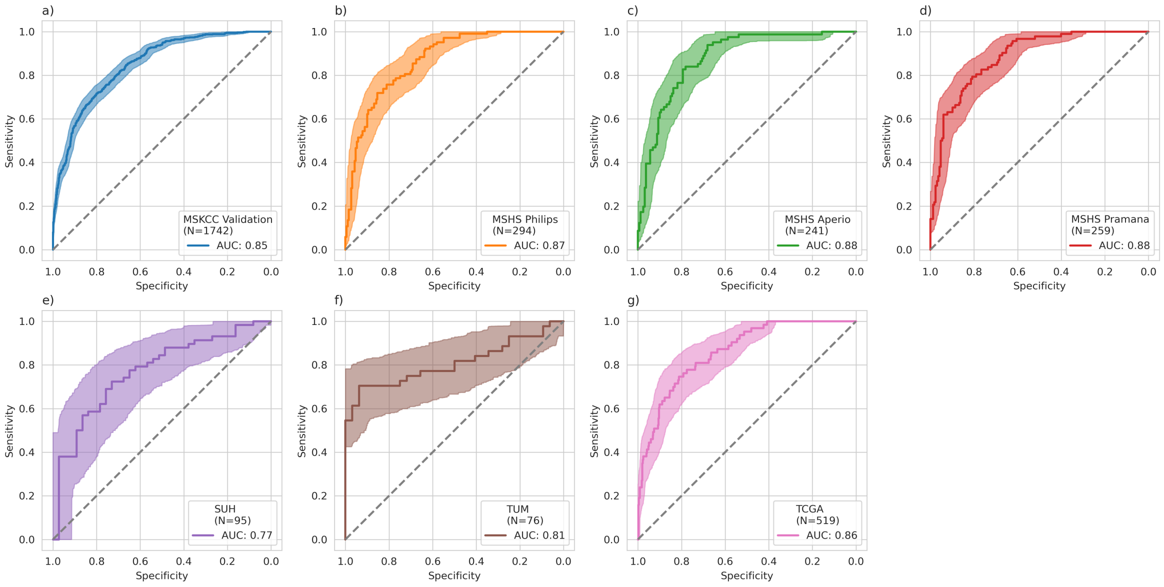

Supp. Figure 1 Validation and test ROC curves comparison. Each test cohort is plotted separately. 95% confidence interval of the ROC curve was computed via bootstrapping with 1,000 samples.

Supp. Table 5 Evaluation of the performance on the internal validation cohort and external test sets. 95% confidence interval (CI) calculated via bootstrapping with 1,000 iterations.

Cohort	N	AUC	95% CI
Internal Validation	1742	0.847	0.828-0.866
External Test	1484	0.870	0.851-0.889
MSHS Philips	294	0.870	0.827-0.907
MSHS Aperio	241	0.877	0.832-0.918
MSHS Pramana	259	0.883	0.843-0.922
SUH	95	0.772	0.672-0.860
TUM	76	0.808	0.708-0.896
TCGA	519	0.860	0.814-0.902

Sample Type Analysis

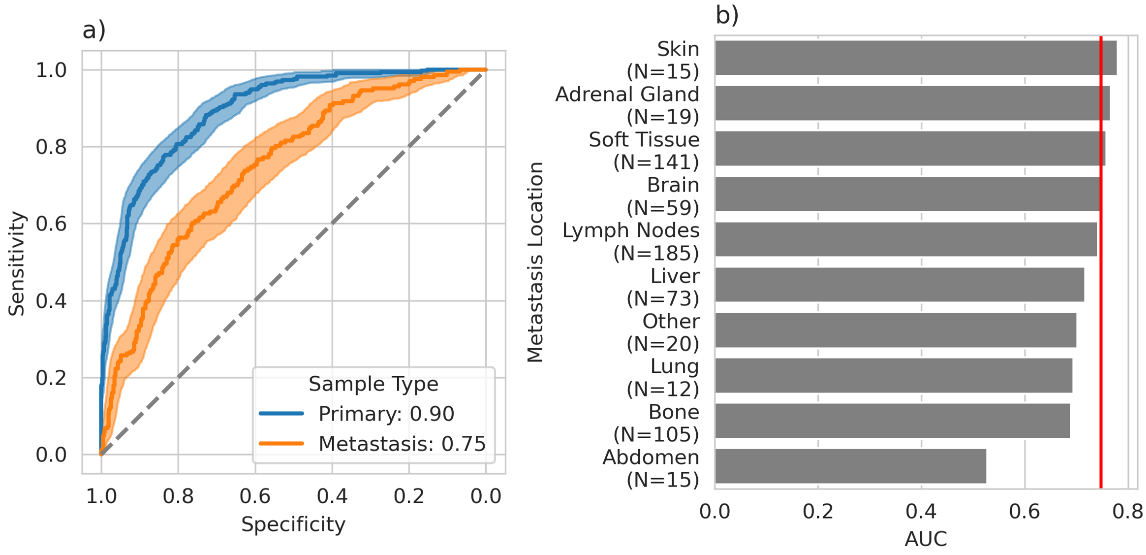

Supp. Figure 2 Analysis of model performance on metastatic samples. a) Internal validation ROC curves stratified by sample type: samples from primary site of disease vs metastatic sites. b) AUC performance on metastatic samples stratified by metastasis location. Each barplot represents a single AUC for each location. The red vertical line represents the overall performance on samples from metastatic sites.

Tissue Area Analysis

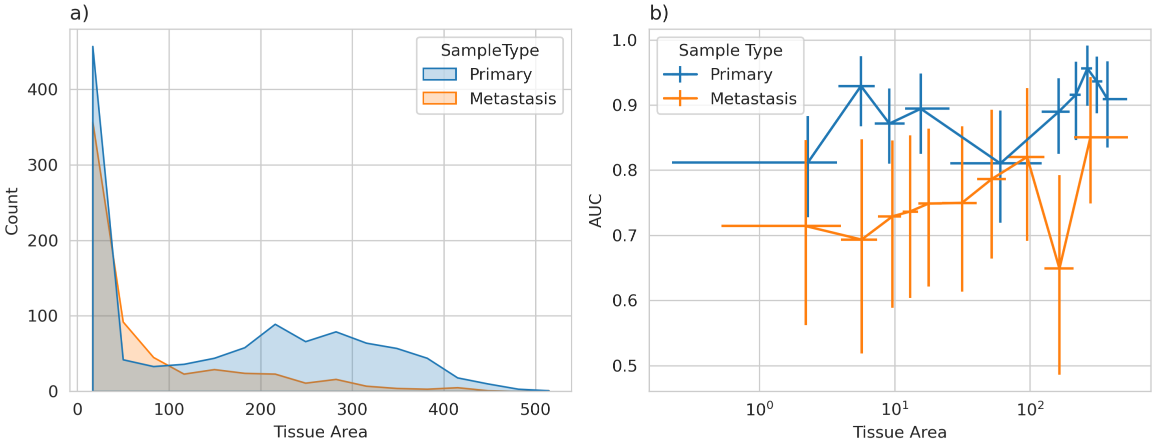

Supp. Figure 3 Analysis of model performance stratified by tissue area (in squared millimeters). a) The Distribution of tissue area per sample for the internal MSKCC validation cohort. b)The distribution of tissue area was divided by deciles. For each bucket the AUC is plotted against the median area of the bucket. The x axis errors bar represents the range of the bucket, while the y axis error bar is the 95% confidence interval estimated via bootstrapping. The analysis was performed for primary and metastatic samples independently.

Supp. Table 6 Analysis of model performance stratified by tissue area (in squared millimeters) for the primary samples in the internal validation cohort. The number of slides and the number of positive slides within each area range is also provided.

Area Range	AUC	Median Area	Slides	*EGFR+*
(0.115, 3.751]	0.812	2.270	111	39
(3.751, 7.125]	0.929	5.639	110	30
(7.125, 11.854]	0.873	9.145	110	38
(11.854, 25.514]	0.895	15.624	110	39
(25.514, 121.84]	0.808	60.443	110	34
(121.84, 196.289]	0.890	163.116	110	39
(196.289, 237.42]	0.915	218.723	110	30
(237.42, 288.236]	0.957	266.102	110	26
(288.236, 344.182]	0.935	312.709	110	30
(344.182, 522.558]	0.910	374.840	110	25

Supp. Table 7 Analysis of model performance stratified by tissue area (in squared millimeters) for the metastatic samples in the internal validation cohort. The number of slides and the number of positive slides within each area range is also provided..

Area Range	AUC	Median Area	Slides	*EGFR+*
(0.391, 4.002]	0.703	2.195	65	14
(4.002, 7.439]	0.693	5.701	64	21
(7.439, 11.164]	0.733	9.659	64	25
(11.164, 14.865]	0.738	12.983	64	20
(14.865, 22.303]	0.749	17.812	64	23
(22.303, 40.354]	0.755	31.479	64	26
(40.354, 66.395]	0.787	52.177	64	25
(66.395, 127.773]	0.824	95.341	64	17
(127.773, 210.601]	0.652	165.405	64	21
(210.601, 531.0]	0.852	280.001	64	14

EGFR Variant Analysis

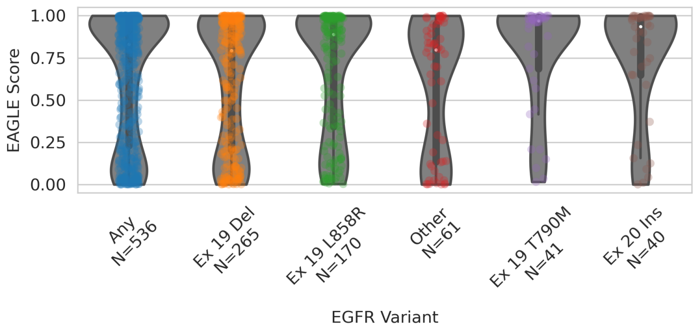

Supp. Figure 4 Comparison of model outputs for different *EGFR* mutation variants.

Supp. Table 8 Statistical significance of score distribution comparisons across mutation variants. Statistical significance was estimated using the 2-sample 2-sided Kolmogorov–Smirnov test. The p-values were corrected using the Bonferroni method.

Sample 1	Sample 2	p-value
Any	Ex 19 Del	9.754
Any	Ex 19 L858R	6.181
Any	Ex 19 T790M	0.033
Any	Ex 20 Ins	1.991
Any	Other	5.276
Ex 19 Del	Ex 19 L858R	1.026
Ex 19 Del	Ex 19 T790M	0.014
Ex 19 Del	Ex 20 Ins	0.450
Ex 19 Del	Other	9.962
Ex 19 L858R	Ex 19 T790M	0.449
Ex 19 L858R	Ex 20 Ins	5.916
Ex 19 L858R	Other	0.967
Ex 19 T790M	Ex 20 Ins	6.473
Ex 19 T790M	Other	0.029
Ex 20 Ins	Other	0.818

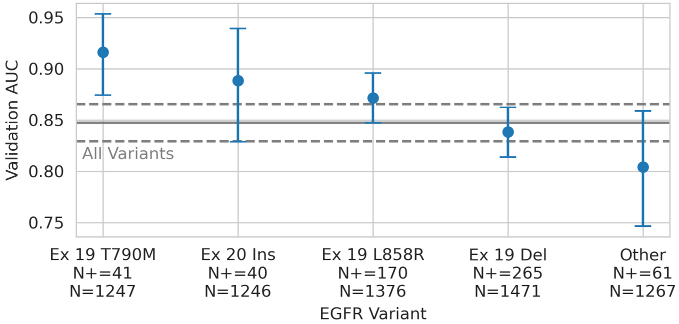

Supp. Figure 5 Validation AUC performance stratified by *EGFR* mutation variant. All variants achieved AUC scores that were not significantly different from the overall AUC score, highlighting the robustness of EAGLE across variants.

MSHS Scanner Analysis

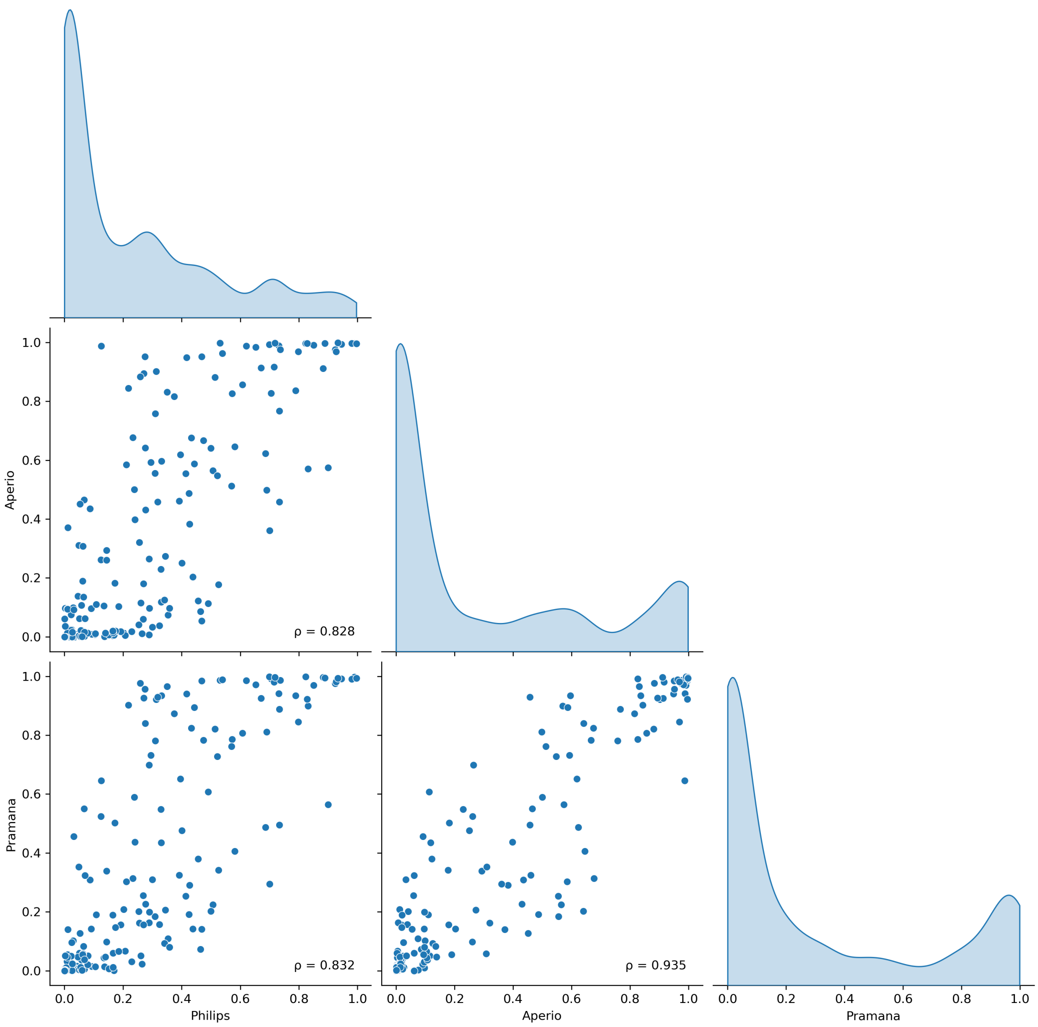

Supp. Figure 6 Comparison of model outputs for paired slides scanned with different scanners. For each comparison, the Pearson correlation coefficient is shown.

Supp. Table 9 Comparison of model outputs for slides scanned with different scanner vendors (N=224). Linear relationship was measured using the Pearson correlation coefficient alongside the calculated p-value for testing non-correlation.

Scanner 1	Scanner 2	r	p-value
Philips	Aperio	0.828	1.3e-57
Philips	Pramana	0.832	1.2e-58
Aperio	Pramana	0.935	5.2e-102

TCGA Artifact Analysis

Supp. Table 10 Summary of slide artifacts for the TCGA dataset curated by a thoracic pathologist.

Artifact	N
Red Saturation	437
Blur	338
Low Quality Stain	325
Freeze Artifacts	265
Severe Artifacts	230
Tissue Necrosis	208
Blue Saturation	63

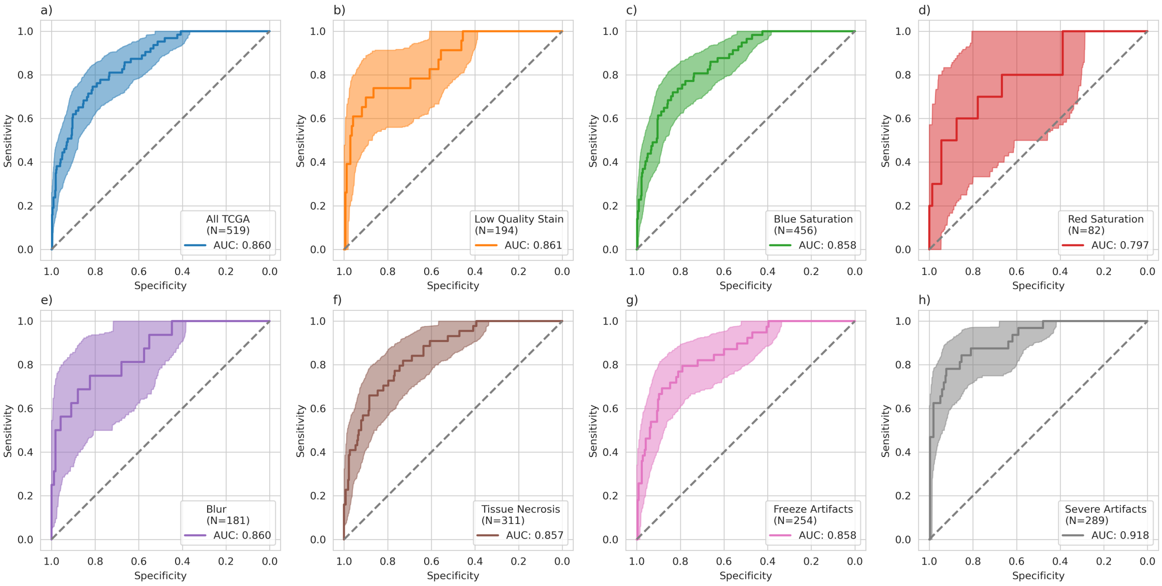

Supp. Figure 7 Model performance results on the overall TCGA cohort and stratified by artifact type. For each artifact type, the result is obtained by removing slides containing the artifact. The shaded ROC region represents the 95% confidence interval calculated via bootstrapping with 1000 iterations.

Turnaround Time

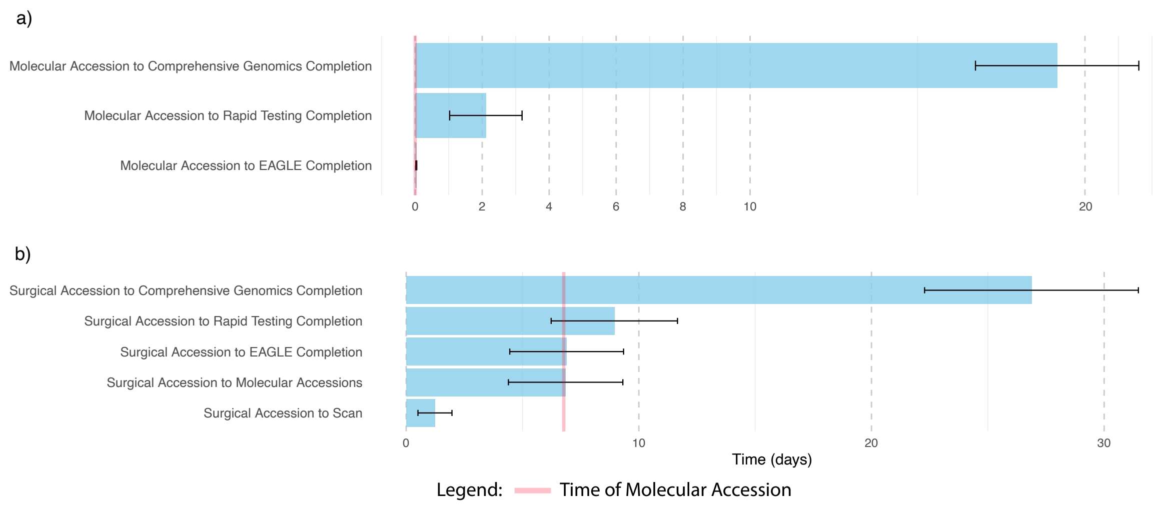

Supp. Figure 8 a) Summary showing the median time from molecular accession to completion of EAGLE, rapid molecular testing, comprehensive genomic sequencing for all samples (N=197) from the silent trial. Error bars show the interquartile range. b) Same as a) but starting from accession of surgical pathology specimen. Also includes time to scanning and time to molecular accession. The red vertical line demonstrates where time zero on plot a) is present on plot b).
