# Supplementary material for: Real-world deployment of a fine-tuned pathology foundation model for lung cancer biomarker detection
Source: Nat Med. 2025 Jul 9;31(9):3002–10. doi: 10.1038/s41591-025-03780-x (PMC12443599; doi:10.1038/s41591-025-03780-x)
Supplement: Supplementary file 2 — Reporting Summary [file 41591_2025_3780_MOESM2_ESM.pdf]

nature portfolio

Corresponding author(s): Chad Vanderbilt, Thomas J. Fuchs

Last updated by author(s): 11/06/2024

Reporting Summary

Nature Portfolio wishes to improve the reproducibility of the work that we publish. This form provides structure for consistency and transparency in reporting. For further information on Nature Portfolio policies, see our Editorial Policies and the Editorial Policy Checklist.

Statistics

For all statistical analyses, confirm that the following items are present in the figure legend, table legend, main text, or Methods section.

n/a	Confirmed	
☐	☒	The exact sample size (*n*) for each experimental group/condition, given as a discrete number and unit of measurement
☐	☒	A statement on whether measurements were taken from distinct samples or whether the same sample was measured repeatedly
☐	☒	The statistical test(s) used AND whether they are one- or two-sided<br>*Only common tests should be described solely by name; describe more complex techniques in the Methods section.*
☐	☒	A description of all covariates tested
☐	☒	A description of any assumptions or corrections, such as tests of normality and adjustment for multiple comparisons
☐	☒	A full description of the statistical parameters including central tendency (e.g. means) or other basic estimates (e.g. regression coefficient) AND variation (e.g. standard deviation) or associated estimates of uncertainty (e.g. confidence intervals)
☐	☒	For null hypothesis testing, the test statistic (e.g. *F*, *t*, *r*) with confidence intervals, effect sizes, degrees of freedom and *P* value noted<br>*Give P values as exact values whenever suitable.*
☒	☐	For Bayesian analysis, information on the choice of priors and Markov chain Monte Carlo settings
☒	☐	For hierarchical and complex designs, identification of the appropriate level for tests and full reporting of outcomes
☐	☒	Estimates of effect sizes (e.g. Cohen's *d*, Pearson's *r*), indicating how they were calculated

Our web collection on statistics for biologists contains articles on many of the points above.

Software and code

Policy information about availability of computer code

Data collection	For digitized slide images, data was directly accessed from institutional databases. Pixel data was accessed using the openslide and cucim libraries. Biomarker results were queried from the molecular laboratory information system at each institution. Data manipulation was performed in python using the pandas library.
Data analysis	We provide the code used for analysis in the GitHub repository linked in the manuscript (https://github.com/chadvanderbilt/EAGLE)

For manuscripts utilizing custom algorithms or software that are central to the research but not yet described in published literature, software must be made available to editors and reviewers. We strongly encourage code deposition in a community repository (e.g. GitHub). See the Nature Portfolio guidelines for submitting code & software for further information.

Data

Policy information about availability of data

All manuscripts must include a data availability statement. This statement should provide the following information, where applicable:

- Accession codes, unique identifiers, or web links for publicly available datasets
- A description of any restrictions on data availability
- For clinical datasets or third party data, please ensure that the statement adheres to our policy

Clinical datasets used for this study are not made available in accordance with institutional policies.

Research involving human participants, their data, or biological material

Policy information about studies with human participants or human data. See also policy information about sex, gender (identity/presentation), and sexual orientation and race, ethnicity and racism.

Reporting on sex and gender	Datasets were not constructed on the basis of sex or gender. We provide aggregate cohort-level sex information as a descriptive statistic.
Reporting on race, ethnicity, or other socially relevant groupings	Datasets were not constructed on the basis of race, ethnicity, or other socially relevant grouping. We provide aggregate cohort-level self-reported race when available as descriptive statistics.
Population characteristics	The relevant population consisted of all lung adenocarcinoma patients with digitized slides and molecular profiling of their cancer. Covariates are reported in tables relevant to each cohort where available: race, sex, age, smoking status, stage, source material and sample type.
Recruitment	NA
Ethics oversight	This research study was approved by the respective Institutional Review Boards at the Icahn School of Medicine at Mount Sinai (Protocol 19-00951) and Memorial Sloan Kettering Cancer Center (Protocol 18-013).

Note that full information on the approval of the study protocol must also be provided in the manuscript.

Field-specific reporting

Please select the one below that is the best fit for your research. If you are not sure, read the appropriate sections before making your selection.

☒ Life sciences ☐ Behavioural & social sciences ☐ Ecological, evolutionary & environmental sciences

For a reference copy of the document with all sections, see nature.com/documents/nr-reporting-summary-flat.pdf

Life sciences study design

All studies must disclose on these points even when the disclosure is negative.

Sample size	Sample size was chosen based on availability. All available samples meeting inclusion criteria were included.
Data exclusions	The tool is developed for lung adenocarcinoma H&E histology. All samples meeting this criteria were included in the study.
Replication	For training generalizability was assessed using the held-out test set strategy. We assessed the generalizability on internal and external test sets (Mt. Sinai, Technical University Munich, Sahlgrenska University Hospital). Performance was also replicated on an entirely separate pre-trial calibration cohort and prospectively in the silent trial. All replication attempts were successful.
Randomization	Randomization into training and testing for the internal data was performed at random and at the patient level.
Blinding	Blinding is not relevant for the development of the model. For the silent trial the results from the model were generate before the genomic results were known. Thus the model is blinded to the EGFR result.

Reporting for specific materials, systems and methods

We require information from authors about some types of materials, experimental systems and methods used in many studies. Here, indicate whether each material, system or method listed is relevant to your study. If you are not sure if a list item applies to your research, read the appropriate section before selecting a response.

Materials & experimental systems

n/a	Involved in the study	
☒	☐	Antibodies
☒	☐	Eukaryotic cell lines
☒	☐	Palaeontology and archaeology
☒	☐	Animals and other organisms
☒	☐	Clinical data
☒	☐	Dual use research of concern
☒	☐	Plants

Methods

n/a	Involved in the study	
☒	☐	ChIP-seq
☒	☐	Flow cytometry
☒	☐	MRI-based neuroimaging

Plants

Seed stocks	*Report on the source of all seed stocks or other plant material used. If applicable, state the seed stock centre and catalogue number. If plant specimens were collected from the field, describe the collection location, date and sampling procedures.*
Novel plant genotypes	*Describe the methods by which all novel plant genotypes were produced. This includes those generated by transgenic approaches, gene editing, chemical/radiation-based mutagenesis and hybridization. For transgenic lines, describe the transformation method, the number of independent lines analyzed and the generation upon which experiments were performed. For gene-edited lines, describe the editor used, the endogenous sequence targeted for editing, the targeting guide RNA sequence (if applicable) and how the editor was applied.*
Authentication	*Describe any authentication procedures for each seed stock used or novel genotype generated. Describe any experiments used to assess the effect of a mutation and, where applicable, how potential secondary effects (e.g. second site T-DNA insertions, mosiacism, off-target gene editing) were examined.*
